# Association between discoid lateral meniscus and medial meniscus posterior root tear: A retrospective cohort study

**DOI:** 10.1002/jeo2.70666

**Published:** 2026-02-18

**Authors:** Ryosuke Yamashita, Yuki Okazaki, Keisuke Kintaka, Koki Kawada, Toshiki Kohara, Takayuki Furumatsu

**Affiliations:** ^1^ Department of Orthopaedic Surgery Okayama University Hospital Okayama Japan; ^2^ Department of Orthopaedic Surgery National Hospital Organization Fukuyama Medical Center Hiroshima Japan; ^3^ Department of Orthopaedic Surgery Kochi Health Sciences Center Kochi Japan; ^4^ Department of Orthopaedic Surgery Japanese Red Cross Okayama Hospital Okayama Japan

**Keywords:** clinical outcome, discoid lateral meniscus, medial meniscus, morphology, posterior root tear

## Abstract

**Purpose:**

Medial meniscus (MM) posterior root tear (PRT) has gained increasing attention because of its biomechanical impact and rapid progression to osteoarthritis. Identifying the risk factors for MMPRT is essential for early diagnosis. Although the discoid lateral meniscus (DLM) is a common congenital variant, no studies have reported on the relationship between MMPRT and DLM. This study aimed to examine the relationship between MMPRT and DLM.

**Methods:**

This retrospective cohort study included 94 matched knees that underwent arthroscopic surgery between 2015 and 2021 (58 with MMPRT and 36 with other MM injuries). The matching was performed according to age and sex. Morphological analysis of the lateral meniscus (LM) was performed using coronal magnetic resonance imaging (MRI). DLM was defined as an LM ratio (LM width/tibial width) > 0.20. LM width, LM ratio and DLM prevalence were compared between the two groups. In the MMPRT group, subgroup analysis compared the preoperative and 1‐year postoperative clinical outcomes between patients with and without DLM.

**Results:**

The PRT group showed significantly greater LM width (12.6 ± 3.1 mm vs. 11.1 ± 2.2 mm; *p* = 0.03) and LM ratio (0.18 ± 0.04 vs. 0.16 ± 0.03; *p* = 0.01) compared with the other group. The incidence of DLM was also significantly higher in the PRT group (29.3% vs. 8.3%; *p* = 0.02). No significant differences in clinical scores were observed between the two subgroups either preoperatively or 1 year postoperatively. However, both groups demonstrated significant improvement in all clinical outcomes 1 year postoperatively (*p* < 0.01).

**Conclusions:**

DLM was significantly more prevalent in patients with MMPRT than in those with other MM injuries. Favourable clinical outcomes were achieved following pullout repair in MMPRT knees regardless of the presence of DLM.

**Level of Evidence:**

Level III, retrospective cohort study.

## Graphical abstract

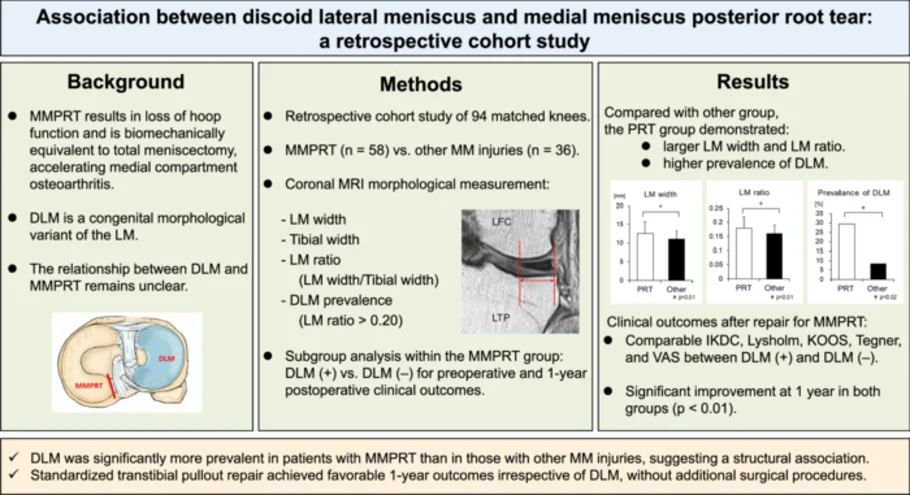

**Abbreviations**
ADL: activities of daily living
BMI: body mass index
CDLM: complete discoid lateral meniscus
DLM: discoid lateral meniscus
ICDLM: incomplete discoid lateral meniscus
ICRS: International Cartilage Repair Society
IKDC: International Knee Documentation Committee
KL: Kellgren–Lawrence
KOOS: knee injury and osteoarthritis outcome score
LM: lateral meniscus
MCID: minimal clinically important difference
MM: medial meniscus
MMPRT: medial meniscus posterior root tear
MRI: magnetic resonance imaging
PASS: patient acceptable symptom state
PROMs: patient‐reported outcome measures
QOL: quality of life
ROM: range of motion
TSS: two simple stitches
VAS: visual analogue scale

## INTRODUCTION

Medial meniscus (MM) posterior root tear (PRT) has gained attention in recent years. The posterior root of the MM plays a critical role in maintaining hoop stress and load distribution across the medial compartment of the knee. MMPRT results in the loss of normal meniscal function, biomechanically equivalent to the effect of total meniscectomy; it is closely associated with rapid progression of medial compartment osteoarthritis [5]. Early diagnosis and treatment are crucial, as MMPRT rapidly accelerates the progression of knee osteoarthritis [11]. Several risk factors for MMPRT have been identified, including advanced age, high body mass index (BMI), varus knee alignment, contralateral MMPRT, a steep posterior tibial slope and shallow medial tibial plateau depth [15]. Additionally, factors such as older age, female sex, obesity (BMI ≥ 30 kg/m²), varus deformity and low levels of sports activity are associated with poorer clinical outcomes following MMPRT repair [32].

The discoid lateral meniscus (DLM) is a congenital morphological variant of the lateral meniscus (LM) that is more frequently observed in Asian populations, with a prevalence ranging from 0.4% to 17% [3]. Although DLM has traditionally been regarded as a lateral pathology, several studies have evaluated limb alignment changes after arthroscopic treatment for DLM, particularly after partial meniscectomy. These findings indicate that surgical intervention of the LM may influence knee biomechanics; however, they do not suggest that an intact DLM itself inherently alters medial compartment loading [13, 31].

In addition to being a potential morphology factor, identifying DLM in patients presenting with knee pain may have direct clinical implications. Earlier recognition of DLM as a possible contributor to MMPRT may prompt clinicians to perform more focused magnetic resonance imaging (MRI) assessment, prioritise early surgical consultation before irreversible joint degeneration occurs. However, no studies have reported a relationship between the DLM and MMPRT, which is a pathology in the medial compartment of the knee. This study aimed to examine the relationship between the MMPRT and DLM. We hypothesised that patients with MMPRT would exhibit a higher prevalence of DLM than those with other types of MM injuries, and that favourable postoperative outcomes could be achieved regardless of the presence or absence of DLM in patients with MMPRT.

## METHODS

### Study design and population

**Figure 1:**
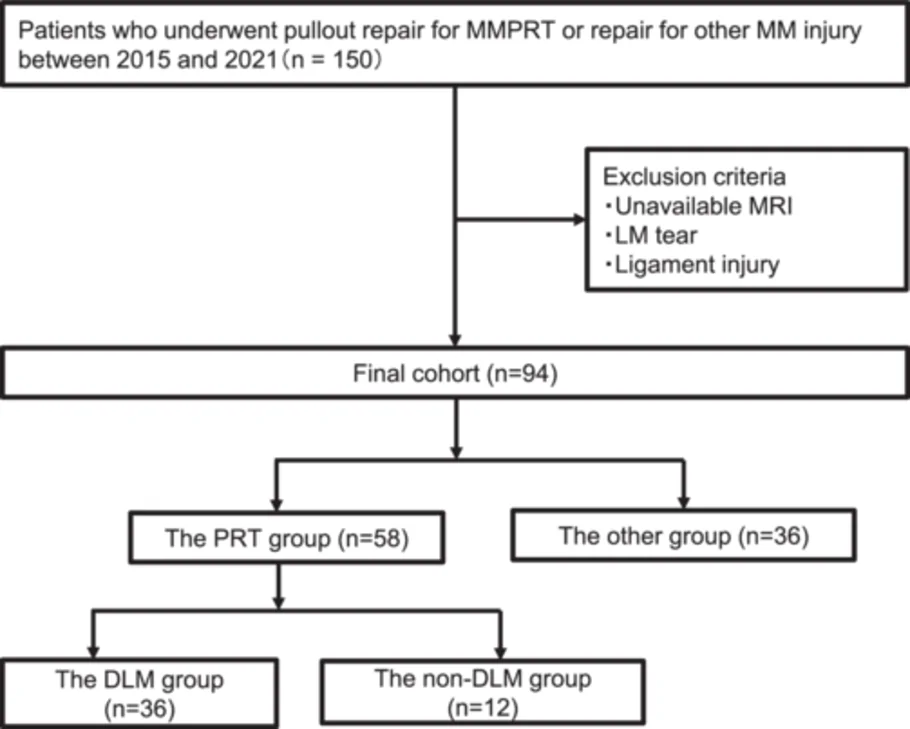
Flowchart of patient recruitment. DLM, discoid lateral meniscus; LM, lateral meniscus; MM, medial meniscus; MMPRT, medial meniscus posterior root tear; MRI, magnetic resonance imaging; PRT, posterior root tear.

This retrospective study included 150 knees (51 male, 99 female; mean age, 63.6 years) that underwent isolated pullout repair for MMPRT or repair for other MM injuries at our institution between 2015 and 2021. The inclusion criteria were: BMI < 30 kg/m^2^, mechanical axis (%MA) > 30, Kellgren–Lawrence (KL) grade < 3 and International Cartilage Repair Society (ICRS) score < 3. Patients were excluded if MRI was unavailable or if concomitant injuries such as LM tears or cruciate ligament injuries were present. A flowchart illustrating patient inclusion and exclusion criteria is presented in Figure 1.

### Surgical procedure and postoperative rehabilitation

**Figure 2:**
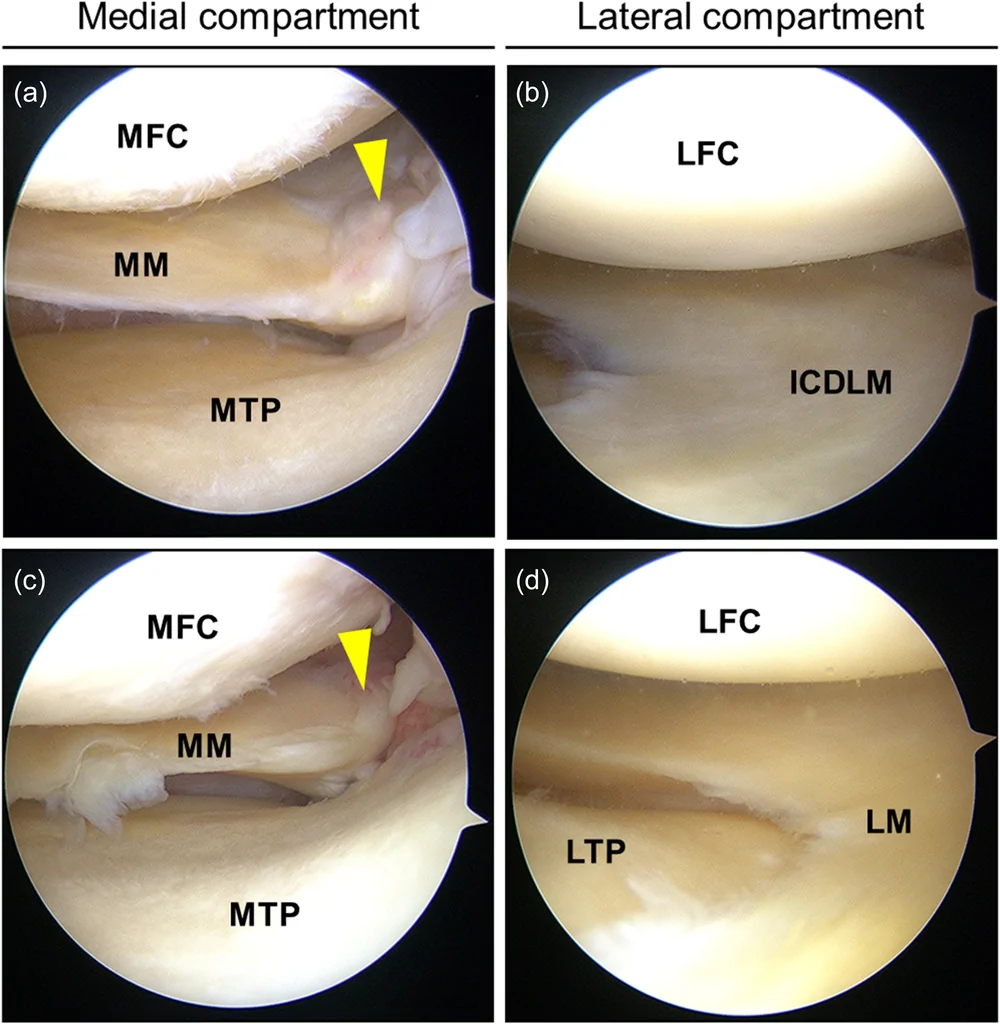
Representative arthroscopic findings of patients with or without discoid lateral meniscus (DLM). (a) The medial compartment of a patient with DLM. The MM posterior root is confirmed (yellow arrowhead). (b) The lateral compartment of a patient with DLM. An incomplete DLM is confirmed. (c) The medial compartment of a patient without DLM. The MM posterior root is confirmed (yellow arrowhead). (d) The lateral compartment of a patient without DLM. No evidence of DLM is identified. ICDLM, incomplete discoid lateral meniscus; LFC, lateral femoral condyle; LM, lateral meniscus; LTP, lateral tibial plateau; MFC, medial femoral condyle; MM, medial meniscus; MTP, medial tibial plateau.

A standard arthroscopic examination was performed using a 4‐mm‐diameter, 30° arthroscope as shown in Figure 2. For patients with a tight medial compartment, the outside‐in pie‐crusting technique was used to facilitate the following procedure [7]. Patients with MMPRT underwent arthroscopic pullout repair using one of three techniques: modified Mason–Allen suture, two simple stitches (TSS), TSS combined with an all‐inside suture to the posteromedial capsule [10]. These sutures were pulled out through the tibial tunnel created with a custom root‐aiming device and fixed to the anterior tibial cortex using a double‐spike plate or bioabsorbable interference screw. In patients with other MM injuries, appropriate procedures were performed according to intraoperative findings, such as inside‐out, all‐inside, outside‐in or a combination of these. Despite concomitant DLM, saucerization was not performed. The asymptomatic and structurally preserved DLM were left untreated.

Postoperative management was standardised. In patients with MMPRT, the knee was immobilised using a brace after surgery. Patients remained non‐weight‐bearing for the first 2 weeks, followed by one‐third partial weight‐bearing from postoperative Week 2, one‐half partial weight‐bearing from Week 3 and full weight‐bearing from Week 4 onward. Range of motion (ROM) was restricted and gradually increased as follows: 0°–45° from Week 2, 0°–60° from Week 3, 0°–90° from Week 4 and 0°–120° from Week 6. Deep knee flexion beyond 120° was allowed after confirmation of root continuity on MRI performed 3 months postoperatively.

In contrast, patients with other MM injuries followed a different rehabilitation protocol. They were kept non‐weight‐bearing for the first two postoperative weeks; however, ROM exercises from 0° to 30° using heel‐slide movements were allowed during this period. From postoperative Week 2, one‐third partial weight‐bearing was permitted with ROM limited to 0°–60°, from Week 3, one‐half partial weight‐bearing with ROM 0°–90° and from Week 4, two‐thirds partial weight‐bearing with ROM 0°–120°. Full weight‐bearing was allowed from postoperative Week 5 onward. Deep knee flexion beyond 120° was permitted 2 months postoperatively.

### Morphological measurements

MRI‐based diagnosis was used to ensure consistent morphological measurements across all patients, while arthroscopic findings were used as supplementary confirmation. Morphological measurements were performed using a preoperative coronal MRI. MRI scans were acquired using a 1.5‐T Achieva system (Philips) equipped with a dedicated knee coil. Coronal T2‐weighted images were obtained with a repetition time (TR) of 637 ms and an echo time (TE) of 18 ms using a 20° flip angle. The slice thickness was 3 mm with a 0.6‐mm interslice gap. The field of view was 16 cm and the acquisition matrix was 205 × 256.

**Figure 3:**
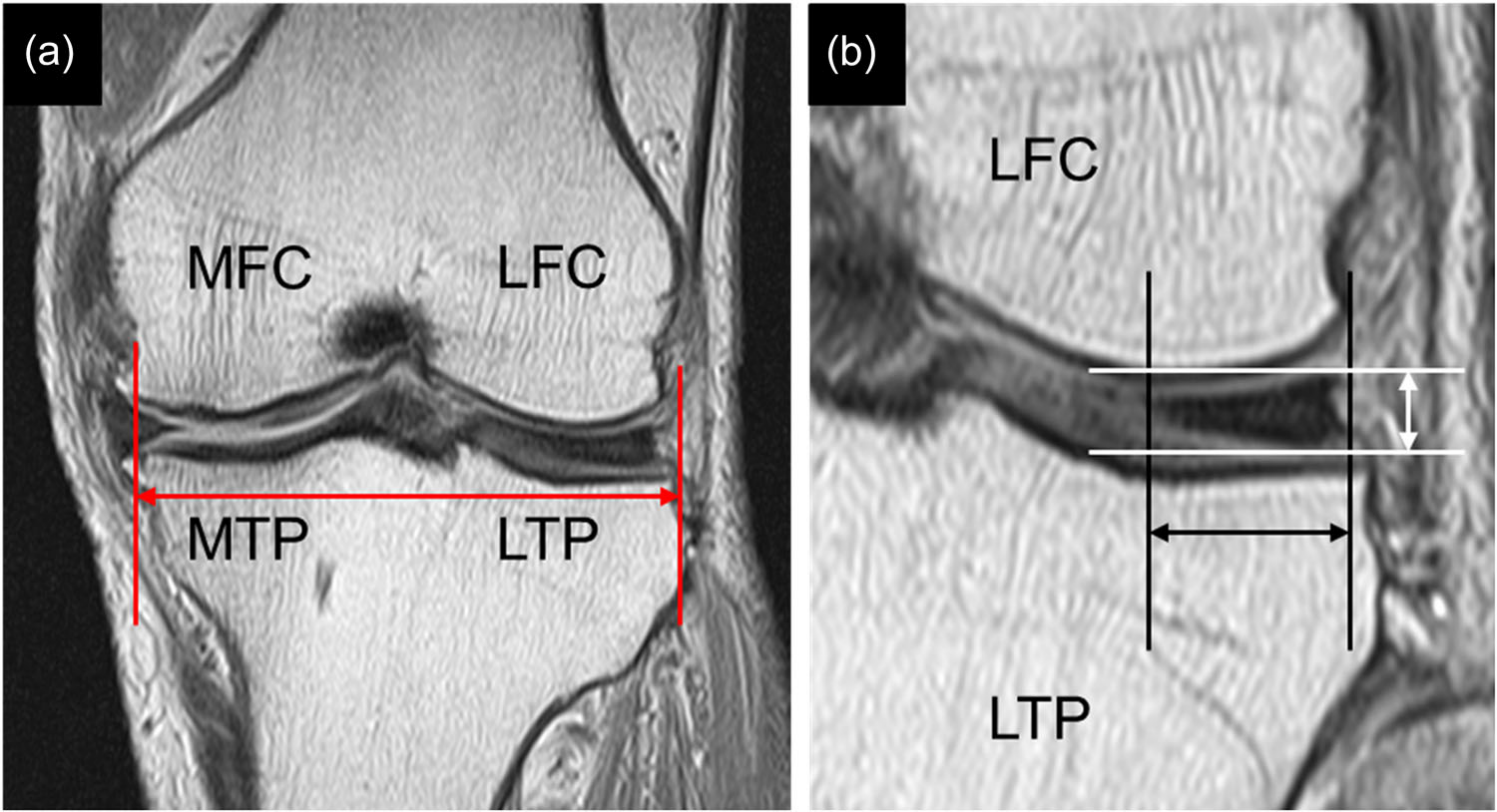
Magnetic resonance imaging‐based measurement of lateral meniscus (LM) morphology on coronal views showing the left knee. (a) The tibial width was defined as the maximum mediolateral distance across the tibia (red double arrow). (b) The LM width, the minimum width of the LM on the slice showing the most prominent meniscus body (black double arrow). LM height, measured vertically from the lowest to highest point of the meniscus (white double arrow). LFC, lateral femoral condyle; LTP, lateral tibial plateau; MFC, medial femoral condyle; MTP, medial tibial plateau.

LM width was defined as the minimum width of the LM, and tibial width was defined as the maximum width of the tibia (excluding osteophytes). The DLM was defined as an LM ratio (LM width divided by tibial width) greater than 0.20, based on the criteria proposed by Samoto et al. [29]. LM height was measured using the method described by Hiranaka et al. [14] (Figure 3). The LM width, tibial width, LM height, LM ratio and incidence of DLM were compared between the PRT and other groups. To assess the reproducibility of morphological measurements, two independent observers each performed the measurements twice on the same images, with a 2‐week interval between the two sessions. Interobserver reliability was evaluated using the intraclass correlation coefficient (ICC [2,1]) based on a two‐way random‐effects model, and intraobserver reliability was assessed using ICC [3,1] based on a two‐way mixed‐effects model.

### Clinical outcomes

Clinical evaluations were performed at the time of arthroscopic surgery and 1 year postoperatively. The following clinical outcomes were assessed: Lysholm score (0 = worst, 100 = best), Tegner activity score (0 = worst, 10 = best), International Knee Documentation Committee (IKDC) score (0 = worst, 100 = best), pain visual analogue scale (VAS; 0 = no pain, 100 = worst possible pain) and knee injury and osteoarthritis outcome scores (KOOS), which comprises five subscales: pain, symptoms, activities of daily living (ADL), sports and recreational function (Sports/Rec) and knee related quality of life (QOL). In each category, 0 was the worst score and 100 was the best [16, 25].

### Subgroup analysis

In the PRT group, patients were further subdivided into two cohorts based on the presence or absence of DLM. Preoperative and 1‐year postoperative clinical outcomes were compared between the groups. The clinical scores included the IKDC score, Lysholm score, Tegner activity score, VAS score for pain and all subscales of the KOOS.

### Statistical analysis

Statistical analysis was performed using EZR software (Saitama Medical Center, Jichi Medical University) [17]. To adjust for baseline differences between groups, propensity score matching was performed based on age and sex. All data were assessed for normality using the Shapiro–Wilk test, and normal distribution was confirmed in all groups. After the analysis, intergroup comparisons were conducted using the *t*‐test, and intragroup differences were analysed using a paired *t*‐test. The incidence of DLM between the groups was compared using Fisher's exact test. Statistical significance was set at *p* < 0.05. Post hoc power analyses were performed for the comparisons of LM width and LM ratio between the PRT and the other groups. The interobserver reliability between the two observers was excellent, with an ICC (2,1) of 0.91. The intraobserver reliability was also excellent, with ICC (3,1) values of 0.94 for observer A and 0.93 for observer B, indicating high reproducibility of the measurements. Based on the observed effect sizes (*d* = 0.52 for LM width and *d* = 0.62 for LM ratio), the statistical power was calculated using a two‐sided *t*‐test with *α* = 0.05. The LM ratio demonstrated adequate power (0.82), whereas the LM width showed insufficient power (0.68).

## RESULTS

After demographic data matching based on age and sex, 94 knees were included and divided into two groups: the PRT group, comprising knees with MMPRT (58 knees: 29 male, 29 female; mean age, 60.4 years), and the other group, comprising knees with other MM injuries (36 knees: 18 male, 18 female; mean age, 61.2 years).

The patient demographics are summarised in Table 1. There were no significant differences between the two groups regarding age, sex, height, weight, BMI, femorotibial angle (FTA) and ％ mechanical axis (％MA).

**Table 1:** Patient demographics and clinical characteristics.

Characteristics	The PRT group	The other group	*p*‐value
Number of patients	58	36	
Sex (male/female)	29/29	18/18	1.0
Age (years)	60.4 ± 8.6	61.2 ± 9.3	0.65
Height (m)	1.62 ± 0.1	1.62 ± 0.1	0.70
Weight (kg)	68.9 ± 15.4	66.7 ± 12.5	0.82
Body mass index (kg/m^2^)	26.2 ± 3.9	25.4 ± 3.7	0.52
Femorotibial angle (°)	177.3 ± 1.9	177.5 ± 1.4	0.69
% mechanical axis (%)	39.1 ± 7.2	38.6 ± 6.7	0.71
Root tear classification (type1/2/4) (*n*)	5/46/7	N/A	

*Note*: Data are presented as mean ± standard deviation or number.

Abbreviations: PRT, posterior root tear; N/A, not applicable.

The MRI‐based morphological comparisons between the PRT and other groups are presented in Table 2. The PRT group demonstrated a significantly greater LM width and LM ratio than the other groups. The incidence of DLM was also significantly higher in the PRT group. No significant differences in tibial width or LM height were observed between the two groups.

**Table 2:** Comparison of magnetic resonance imaging‐based morphological measurements and incidence of DLM between the PRT and the other group.

	The PRT group	The other group	*p*‐value
LM width (mm)	12.6 ± 3.1	11.1 ± 2.2	0.03*
Tibial width (mm)	70.1 ± 6.2	71.0 ± 5.9	0.42
LM ratio	0.18 ± 0.04	0.16 ± 0.03	0.01*
LM height (mm)	7.06 ± 1.14	7.05 ± 1.23	0.69
Incidence of DLM	17/58 (29.3%)	3/36 (8.3%)	0.02*

*Note*: Data are presented as mean ± standard deviation or number (%). The LM ratio was defined as the LM width divided by the tibial width. DLM was defined as an LM ratio > 0.20 according to the criteria proposed by Samoto et al. [29].

Abbreviations: DLM, discoid lateral meniscus; LM, lateral meniscus; PRT, posterior root tear.

* Statistically significant differences were analysed using *t*‐test (*p* < 0.05).

The demographic characteristics of the DLM and non‐DLM subgroups within the MMPRT group are summarised in Table 3. There were no significant differences in age, sex, height, weight or BMI between the two subgroups. No significant differences were observed in the KOOS subscales, Lysholm score, IKDC score, Tegner activity score or VAS pain scale between the DLM and non‐DLM groups both preoperatively and 1‐year postoperatively (Tables 4 and 5). Intragroup analysis revealed significant improvements in all clinical scores at 1 year postoperatively compared to the preoperative scores in both the DLM and non‐DLM groups (Supporting Information: Tables S1 and S2; *p* < 0.01 for all).

**Table 3:** Patient demographics and clinical characteristics in patients with and without DLM in the posterior root tear group.

Characteristics	DLM group	non‐DLM group	*p*‐value
Number of patients	17	17	
Sex (male/female)	9/8	8/9	1.0
Age (years)	61.5 ± 7.4	62.0 ± 8.9	0.85
Height (m)	1.59 ± 0.1	1.62 ± 0.07	0.35
Weight (kg)	64.1 ± 11.1	66.4 ± 14.0	0.61
Body mass index (kg/m^2^)	25.1 ± 3.1	25.1 ± 4.0	0.98

*Note*: Data are presented as mean ± standard deviation or number.

Abbreviation: DLM, discoid lateral meniscus.

**Table 4:** Preoperative clinical scores in patients with and without DLM in the posterior root tear group.

	DLM group	non‐DLM group	*p*‐value
KOOS			
– ADL	23.1 ± 12.6	32.1 ± 20.3	0.15
– Pain	56.1 ± 10.5	51.5 ± 22.5	0.49
– Symptoms	58.3 ± 17.7	60.2 ± 20.8	0.79
– QOL	68.3 ± 9.1	63.4 ± 19.4	0.45
– Sports/Rec	21.9 ± 21.3	27.8 ± 26.9	0.45
Lysholm score	56.5 ± 4.8	59.4 ± 8.9	0.95
IKDC score	36.1 ± 11.8	38.2 ± 16.6	0.69
Tegner activity score	1.33 ± 0.78	1.35 ± 0.79	0.95
Visual analogue scale pain	33.7 ± 25.9	43.8 ± 27.5	0.27

*Note*: Data are presented as mean ± standard deviation. No statistically significant intergroup differences were detected for any of the parameters (all *p* > 0.05).

Abbreviations: ADL, activities of daily living; DLM, discoid lateral meniscus; KOOS, Knee Injury and Osteoarthritis Outcome Score; IKDC, International Knee Documentation Committee score; QOL, quality of life; Sports/Rec, sports and recreational function.

**Table 5:** One‐year postoperative clinical outcome scores in patients with and without DLM in the posterior root tear group.

	DLM	non‐DLM	*p*‐value
KOOS			
– ADL	65.7 ± 15.5	58.3 ± 17.3	0.19
– Pain	88.9 ± 6.98	81.9 ± 12.4	0.07
– Symptoms	80.7 ± 11.9	72.9 ± 13.9	0.09
– QOL	88.1 ± 8.09	83.9 ± 11.3	0.24
– Sports/Rec	51.7 ± 26.2	46.1 ± 27.0	0.19
Lysholm score	87.6 ± 4.6	85.7 ± 6.5	0.35
IKDC score	67.1 ± 10.1	61.9 ± 12.2	0.19
Tegner activity score	3.25 ± 0.45	2.89 ± 0.66	0.09
Visual analogue scale pain	8.67 ± 7.56	12.8 ± 11.3	0.25

*Note*: Data are presented as mean ± standard deviation. No statistically significant intergroup differences were detected for any parameter (all *p* > 0.05).

Abbreviations: ADL, activities of daily living; DLM, discoid lateral meniscus; KOOS, Knee Injury and Osteoarthritis Outcome Score; IKDC, International Knee Documentation Committee score; QOL, quality of life; Sports/Rec, sports and recreational function.

## DISCUSSION

The most important finding of this study was that patients with MMPRT had a significantly higher incidence of DLM and exhibited a larger LM width and LM ratio than patients with other MM injuries. These findings indicate an association between DLM and MMPRT. Importantly, this association should be interpreted as a morphological and diagnostic observation rather than risk factor. Although the LM ratio demonstrated sufficient statistical power, the comparison of LM width was underpowered due to the limited sample size, particularly in the other group. This should be considered when interpreting the negative findings regarding LM width.

DLM is a congenital morphological variant most frequently observed in LM, with a reported prevalence ranging from 0.4% to 17% in Asian cohorts [3]. However, the true prevalence is likely to be underestimated because of the high proportion of asymptomatic cases. A population‐based study reported a prevalence of 4.88 per 100,000 individuals, with no significant association with ethnicity, sex or BMI [12]. Bilateral involvement is common and is seen in 15%–25% of symptomatic Asian patients [4].

Developmentally, DLM is believed to result from incomplete resorption of the central meniscus during fetal development, leaving behind a residual prenatal morphology [6, 20]. Several anatomical and histological characteristics of DLM have been proposed to explain its susceptibility to meniscal tears. Morphologically, the DLM is thicker and wider than a normal LM and often lacks a normal crescent shape and posterior horn tapering. These features can result in abnormal stress distribution and increased mechanical impingement during knee motion [1, 19]. These characteristics may predispose the DLM to tearing, particularly under abnormal loading conditions.

Historically, total or subtotal meniscectomy was commonly performed for symptomatic DLM, but this approach has been associated with an increased risk of early lateral compartment osteoarthritis [2]. Currently, the standard treatment for symptomatic DLM is arthroscopic saucerization with the preservation of an approximately 8 mm peripheral rim, which helps maintain hoop stress function and joint congruity [9, 30]. Meniscal repair using suturing techniques is recommended in patients with peripheral instability. Clinical outcomes of saucerization combined with repair have been favourable in both paediatric and adult populations, yielding good functional recovery and minimising degenerative progression [27, 28].

Previous studies have reported altered lower‐limb alignment and an increased prevalence of varus deformity in patients with torn or surgically treated DLM [13, 21, 22]. However, these findings cannot be directly extrapolated to patients with intact DLM. Notably, in the present study, no significant differences in lower‐limb alignment parameters were observed between the PRT group and the other group, further suggesting that previously reported alignment changes may not be directly applicable to intact DLM.

Lower‐limb alignment is a well‐established determinant of medial compartment loading and a recognised risk factor for MMPRT [23]. However, the absence of significant alignment differences between the two groups in the present study suggests that the observed association between DLM and MMPRT cannot be explained solely by differences in coronal alignment. From this perspective, DLM should be regarded not as a primary biomechanical driver, but rather as a morphological background factor that may be associated with the biomechanical environment of the knee.

Transtibial pullout repair has been demonstrated to reliably improve patient‐reported outcome measures (PROMs), including the IKDC, KOOS and Lysholm scores, at the 5‐year follow‐up, with low reoperation and arthroplasty conversion rates [26]. Moreover, anatomical techniques have been shown to optimise meniscal healing and reduce MM extrusion compared to non‐anatomical methods [18]. Given that DLM may alter the medial compartment loading patterns and predispose patients to MMPRT, early surgical intervention may be particularly beneficial in preserving meniscal hoop function and delaying degenerative changes. Recent evidence indicates that pullout repair restores tibiofemoral contact mechanics to normal levels, thereby mitigating the adverse effects of MMPRT [8].

Maheshwer et al. established thresholds for the minimal clinically important difference (MCID) and patient‐acceptable symptomatic state (PASS) for PROMs following arthroscopic meniscal repair. They reported MCID thresholds of 10.9 for the IKDC score and 12.3, 11.8, 11.4, 16.7 and 16.9 for the KOOS Symptoms, Pain, ADL, Sport and QOL subscales, respectively, and demonstrated that achieving these thresholds is associated with clinically meaningful recovery [24]. In the present study, postoperative improvements in IKDC, KOOS exceeded the reported MCID values, and the mean postoperative scores fell within the PASS ranges in both subgroups. These findings suggest that transtibial pullout repair for MMPRT provides clinically meaningful and patient‐acceptable symptom relief, even in patients with DLM.

From a clinical relevance, identification of concomitant DLM may heighten diagnostic awareness, prompting careful preoperative MRI assessment and meticulous intraoperative inspection of the MM posterior root. Importantly, despite the presence of intact DLM, favourable clinical outcomes were achieved with transtibial pullout repair alone, and our findings do not support the need for additional alignment‐correcting procedures, such as osteotomy.

This study had several limitations. First, owing to the relatively limited sample size, propensity score matching was performed using only age and sex. However, there were no statistically significant differences in other demographic variables between the groups. Second, although key morphological parameters were evaluated and strict inclusion criteria were applied, other potential risk factors for MMPRT were not quantitatively assessed. Third, owing to the cross‐sectional nature of this study, a definitive causal relationship between DLM and MMPRT could not be established. Longitudinal studies are required to determine whether the DLM contributes to or precedes the development of MMPRT. However, given the high prevalence of asymptomatic DLM, conducting prospective studies is challenging. Fourth, because the follow‐up period was limited to 1 year, the long‐term impact of DLM morphology on the prognosis of MMPRT may not have been fully evaluated. Future studies with extended follow‐up are required to assess both clinical outcomes and structural changes over time. Finally, the morphological analysis was based solely on MRI parameters; dynamic biomechanical assessments, such as gait analysis or weight‐bearing MRI, were not performed. Future research should also explore the biomechanical effects of the DLM on medial compartment loading using tools such as gait analysis and finite element modelling.

## CONCLUSIONS

Patients with MMPRT demonstrated a significantly higher prevalence of DLM and a greater LM ratio compared with patients with other MM injuries, indicating an association between DLM and MMPRT. This association should be interpreted as a morphological and diagnostic finding rather than evidence of a causal relationship, particularly given that no significant differences in lower‐limb alignment were observed between groups. Arthroscopic transtibial pullout repair resulted in clinically meaningful improvements in PROMs regardless of the presence of intact DLM, without the need for additional surgical intervention targeting the lateral compartment. These findings suggest that recognition of DLM may aid diagnostic awareness in patients with MMPRT, while standardised anatomical pullout repair remains an effective treatment strategy.

## AUTHOR CONTRIBUTIONS

Takayuki Furumatsu contributed to the study conception and design. Ryosuke Yamashita and Keisuke Kintaka performed the data collection and analysis. Ryosuke Yamashita and Yuki Okazaki drafted the manuscript. All authors have critically revised and approved the final version.

## CONFLICT OF INTEREST STATEMENT

The authors declare no conflicts of interest.

## ETHICS STATEMENT

This study was approved by the Institutional Review Board in Okayama University (Ethical approval No.1857). Written informed consent was obtained from all study participants.

## LANGUAGE EDITING AND USE OF ARTIFICIAL INTELLIGENCE

The manuscript was professionally edited for English language usage, grammar and clarity by a native English‐speaking medical editor (Editage). In addition, an AI‐based language model was used to assist with manuscript refinement. The authors take full responsibility for the content of the manuscript.

## Supporting information

Supporting information.

Supporting information.

## Data Availability

Upon reasonable request, data supporting the findings of this study are available from the corresponding author.
