# Supplementary material for: Association between discoid lateral meniscus and medial meniscus posterior root tear: A retrospective cohort study
Source: J Exp Orthop. 2026 Feb 18;13(1):e70666. doi: 10.1002/jeo2.70666 (PMC12914482; doi:10.1002/jeo2.70666)
Supplement: Supplementary file 1 — Supporting information. [file JEO2-13-e70666-s001.docx]

**Suppl 1. Intragroup comparison of preoperative and one-year postoperative clinical scores in patients with discoid lateral meniscus in the posterior root tear group**

|  | Preoperative Score | 1-Year Score | P value |
| --- | --- | --- | --- |
| KOOS – ADL | 68.3 ± 9.1 | 88.1 ± 8.1 | P<0.01 |
| – Pain | 56.2 ± 10.5 | 88.9 ± 7.0 | P<0.01 |
| – Symptoms | 23.1 ± 12.6 | 65.8 ± 15.5 | P<0.01 |
| – QOL | 21.3 ± 22.0 | 51.7 ± 26.2 | P<0.01 |
| – Sports/Rec | 58.3 ± 17.7 | 80.7 ± 11.9 | P<0.01 |
| Lysholm score | 36.2 ± 11.9 | 67.2 ± 10.1 | P<0.01 |
| IKDC score | 56.5 ± 4.8 | 87.7 ± 4.6 | P<0.01 |
| Tegner activity score | 1.33 ± 0.78 | 3.25 ± 0.45 | P<0.01 |
| Visual analogue scale pain | 33.8 ± 26.0 | 8.7 ± 7.6 | P<0.01 |

**Suppl 2. Intragroup comparison of preoperative and one-year postoperative clinical scores in patients without discoid lateral meniscus in the posterior root tear group**

|  | Preoperative Score | 1-Year Score | p-value |
| --- | --- | --- | --- |
| KOOS – ADL | 63.4 ± 19.4 | 83.9 ± 11.3 | P<0.01 |
| – Pain | 51.5 ± 22.5 | 82.0 ± 12.4 | P<0.01 |
| – Symptoms | 32.1 ± 20.3 | 58.3 ± 17.3 | P<0.01 |
| – QOL | 27.8 ± 26.9 | 46.1 ± 27.1 | P<0.01 |
| – Sports/Rec | 60.2 ± 20.9 | 72.9 ± 13.9 | P<0.01 |
| Lysholm score | 38.3 ± 16.6 | 62.0 ± 12.3 | P<0.01 |
| IKDC score | 59.4 ± 8.9 | 85.7 ± 6.5 | P<0.01 |
| Tegner activity score | 1.35 ± 0.79 | 2.89 ± 0.66 | P<0.01 |
| Visual analogue scale pain | 43.8 ± 27.6 | 12.8 ± 11.3 | P<0.01 |
