# Supplementary material for: Association between discoid lateral meniscus and medial meniscus posterior root tear: A retrospective cohort study
Source: J Exp Orthop. 2026 Feb 18;13(1):e70666. doi: 10.1002/jeo2.70666 (PMC12914482; doi:10.1002/jeo2.70666)
Supplement: Supplementary file 2 — Supporting information. [file JEO2-13-e70666-s002.docx]

**Supplementary table legends**

**Suppl 1. Intragroup comparison of preoperative and one-year postoperative clinical scores in patients with discoid lateral meniscus in the posterior root tear group**

Data are presented as mean ± standard deviation. All parameters significantly improved postoperatively compared with the preoperative values, as assessed by the paired t-test (P < 0.01 all).

ADL, activities of daily living; KOOS, Knee Injury and Osteoarthritis Outcome Score; IKDC, International Knee Documentation Committee score; QOL, quality of life; Sports/Rec, sports and recreational function.

**Suppl 2. Intragroup comparison of preoperative and one-year postoperative clinical scores in patients without discoid lateral meniscus in the posterior root tear group**

Data are presented as mean ± standard deviation. All parameters showed significant improvement at 1-year compared with baseline, according to the paired t-test analysis (p < 0.01 for all).

ADL, activities of daily living; KOOS, Knee Injury and Osteoarthritis Outcome Score; IKDC, International Knee Documentation Committee score; QOL, quality of life; Sports/Rec, sports and recreational function.
